# Evaluating COVID-19 control measures in mass gathering events with vaccine inequalities

**DOI:** 10.1038/s41598-022-07609-2

**Published:** 2022-03-07

**Authors:** Ali M. Al-Shaery, Bilal Hejase, Abdessamad Tridane, Norah S. Farooqi, Hamad Al Jassmi

**Affiliations:** 1 Department of Civil Engineering, College of Engineering and Islamic Architecture, Umm Al-Qura University, Makkah, Saudi Arabia; 2 Department of Electrical Engineering, Ohio State University, Columbus, OH 43210 USA; 3 Mathematical Sciences Department, College of Science, United Arab Emirates University, Al Ain, UAE; 4 College of Computer and Information Systems, Umm Al-Qura University, Makkah, Saudi Arabia; 5 Emirates Center for Mobility Research, United Arab Emirates University, Al Ain, UAE; 6 Department of Civil and Environmental Engineering, College of Engineering, United Arab Emirates University, Al Ain, UAE

**Keywords:** Mathematics and computing, Viral infection, Public health

## Abstract

With the increasing global adoption of COVID-19 vaccines, limitations on mass gathering events have started to gradually loosen. However, the large vaccine inequality recorded among different countries is an important aspect that policymakers must address when implementing control measures for such events. In this paper, we propose a model for the assessment of different control measures with the consideration of vaccine inequality in the population. Two control measures are considered: selecting participants based on vaccine efficacy and restricting the event capacity. We build the model using agent-based modeling to capture the spatiotemporal crowd dynamics and utilize a genetic algorithm to assess the control strategies. This assessment is based on factors that are important for policymakers such as disease prevalence, vaccine diversity, and event capacity. A quantitative evaluation of vaccine diversity using the Simpson’s Diversity Index is also provided. The Hajj ritual is used as a case study. We show that strategies that prioritized lowering the prevalence resulted in low event capacity but facilitated vaccine diversity. Moreover, strategies that prioritized diversity resulted in high infection rates. However, increasing the prioritization of participants with high vaccine efficacy significantly decreased the disease prevalence. Strategies that prioritized ritual capacity did not show clear trends.

## Introduction

The spread of COVID-19 has been highly attributed to high population densities and the rate of contacts between individuals^1^. Examples of such events include the Diamond Princess cruise ship or mass gathering events such as the Hajj and the Olympic Games. These conditions also provide the possibility for superspreading events that can be traced back to a significant number of COVID-19 cases^2^. For instance, in the isolated superspreader event on the Diamond Princess cruise ship, 634 positive cases were recorded from a single cluster^3^. Moreover, one study attributes the spread of COVID-19 in South Korea to a church cluster that resulted in more than 3900 secondary cases , which accounted for 55% of the cases at the time^4^. Several other events were also recorded that represented superspreader events in religious gatherings, food processing plants, work, hospitals, elderly care centers, and schools^2^.

To limit such events and reduce the effect of the pandemic, various control measures have been put in place such as banning mass gatherings, enforcing social-distancing requirements, and—more recently—requiring vaccinations. While vaccinations provide hope for a return to normalcy for mass gathering events, the rise of new variants of COVID-19 that are capable of both spreading faster and undermining vaccine efficacy^5^ require event planners to carefully consider the implemented control strategies. For global mass gathering events such as the Hajj and the Olympic Games, an effective and equal vaccine rollout is necessary to curb the scope of infection events. However, the global rollout of vaccines shows great inequalities between high and medium-income countries and low-income countries^6^. This results in large vaccination differences between different countries that would require policymakers to place more restrictions from countries with low vaccination rates. In this paper, we propose an analysis method for policymakers to assess effective control strategies that take into consideration the unequal distribution of vaccines. We refer to the distribution of different vaccines as the vaccine diversity. As a case study, we consider the Hajj ritual as it represents the largest mass gathering event with global participants. More specifically, we limit the scope of the Hajj to the Tawaf: one of the rituals that presents a high crowd density scenario with spatiotemporal requirements.

The Hajj pilgrimage is an annual Islamic pilgrimage to Makkah (Mecca) in Saudi Arabia. It is considered one of the five pillars of Islam and is compulsory for those that are physically fit and able to afford it. The Tawaf is a part of the Hajj pilgrimage where pilgrims circle the outside of the Kaaba counterclockwise seven times in an area called the Mataf. For the past ten years prior to 2020, the Hajj has almost consistently drawn more than two million pilgrims from over a hundred countries from all continents^7^. As such, implications of a coronavirus (COVID-19) infection event extend not only to the pilgrims and the local population but to the global population as well. Due to the international diversity of the participating population, it is necessary for policymakers to consider the effect of uneven global vaccine rollout when assessing effective control strategies.

The high crowd density of the rituals of Hajj provides an environment for the spread of respiratory diseases. Such diseases have been found to be the most frequent with an estimated 1 in every 3 pilgrims experiencing respiratory syndromes^8–10^. One study of French Hajj pilgrims has shown that more than 80% reported at least one respiratory syndrome during the 2014–2017 Hajj seasons^11^. In recent years, health policy officials have been challenged with disease outbreaks such as the Severe Acute Respiratory Syndrome (SARS), Middle East Respiratory Syndrome (MERS-CoV), and Ebola^12^. Considerable effort has been put by policymakers to enforce measures that address these challenges such as vaccination requirements for pilgrims against yellow fever, meningitis, and polio^13^; continuous monitoring of emerging diseases and proactive surveillance of communicable diseases; and deployment of information technology and health surveillance systems for quick planning and response^14^.

Tools for assessing effective control strategies are essential for determining the right combination of measures^15^. Agent-based models (ABM) have been proposed to study the effect of control measures such as face masks, screening, and population control^16^ . These models have also been used to estimate risky contacts and study the effect of physical distancing measures on the Hajj pilgrims during the rituals^17^. Another line of research investigates autoregressive models to forecast the spread of the pandemic^18,19^. Epidemic compartmental models^20^ have also been proposed to estimate the progression of COVID-19^21^ and to measure the impact of preventative measures such as suspending Umrah, which is another Islamic pilgrimage^22^. While these studies aim to model and forecast the spread of disease within the Hajj, they do not consider the effect of vaccinations and the global vaccine inequality on an agent-based level. Successful control policies for returning to normalcy need to expand beyond conventional measures, such as requiring facemasks or social distancing, and adapt to the global adoption of vaccines.

Studies of the spatial propagation of COVID-19 have also been conducted on a global level. One study highlights the differences in the incidence of COVID-19 between urban and rural areas of the United States and notes that rural areas tend to have a higher concentration of cases^23^. Similarly, spatiotemporal patterns of the spread of COVID-19 have been studied in Brazil^24^, China^25^, England^26,27^, and the United States^28^. Such studies used collected data on the pandemic to find patterns between spatial locations and infection hotspots and the evolution of these hotspots over time. These patterns are useful for determining appropriate allocation of resources to reduce the incidence rate across a spatial geometry. Moreover, metric geometry approaches have been used to study the geographical dispersion of COVID-19 using spatiotemporal data^29^. While the aforementioned studies made valuable contributions for determining the macro-spatial features that drives the spread of COVID-19, this study rather focuses on the micro-spatial propagation of COVID-19 at a much smaller scale, such as the Mataf geometry. We model the spatiotemporal propagation of the disease using the event spatial geometry and the crowd dynamics.

In this paper, we propose an ABM model of the Tawaf ritual and a genetic algorithm approach to find appropriate control strategies under different evaluation metrics that policymakers may consider. These evaluation metrics include the disease prevalence, vaccine diversity, and the event capacity. To our knowledge, there has been no study that simulated different control strategy scenarios for the COVID-19 pandemic under the assumption of vaccinations using ABMs and with consideration to vaccine diversity within the population. The proposed model incorporates the effect of different vaccine efficacy by classifying the population based on the vaccine protection that a pilgrim may have. The model also leverages the Simpson’s Diversity Index^30^ to quantify the level of vaccine diversity and to assess the effect of different control strategies towards addressing the issue of global vaccine inequality. While the Hajj is used as a case study, the proposed methods can be extended to any mass gathering event. Such assessments are critical for the implementation of effective control measures that can contribute to the return to normalcy for mass gathering events.

## Methods

### Simulation environment

We simulate the transmission of a COVID-19 infection event in the Tawaf ritual using an agent-based model developed in NetLogo^31^. The environment is represented as a grid with each grid cell called a “patch”. The model considers the unique spatial constraints induced by the proceedings of the ritual by constraining the traversable patches for the agents to that of the Mataf geometry, the area surrounding the Kaaba.

In general, the agents are motivated to move towards the center of the Mataf in order to perform seven counterclockwise rotations and complete the ritual. Once an agent completes the ritual, they are removed from the simulation. New agents are introduced at each step while not exceeding the total ritual capacity defined. A set of behavioral rules, together with the spatial constraints, induce a realistic and natural crowd behavior:

- Agents are randomly placed outside the walls of the Mataf area and enter through the nearest gate.
- Agents move slightly outwards when in a crowded area in order to induce intermixing within the crowd.
- When such a move is feasible, agents move such that the next position is closer to the goal while also avoiding collisions with the environment and other agents.

**Figure 1:**
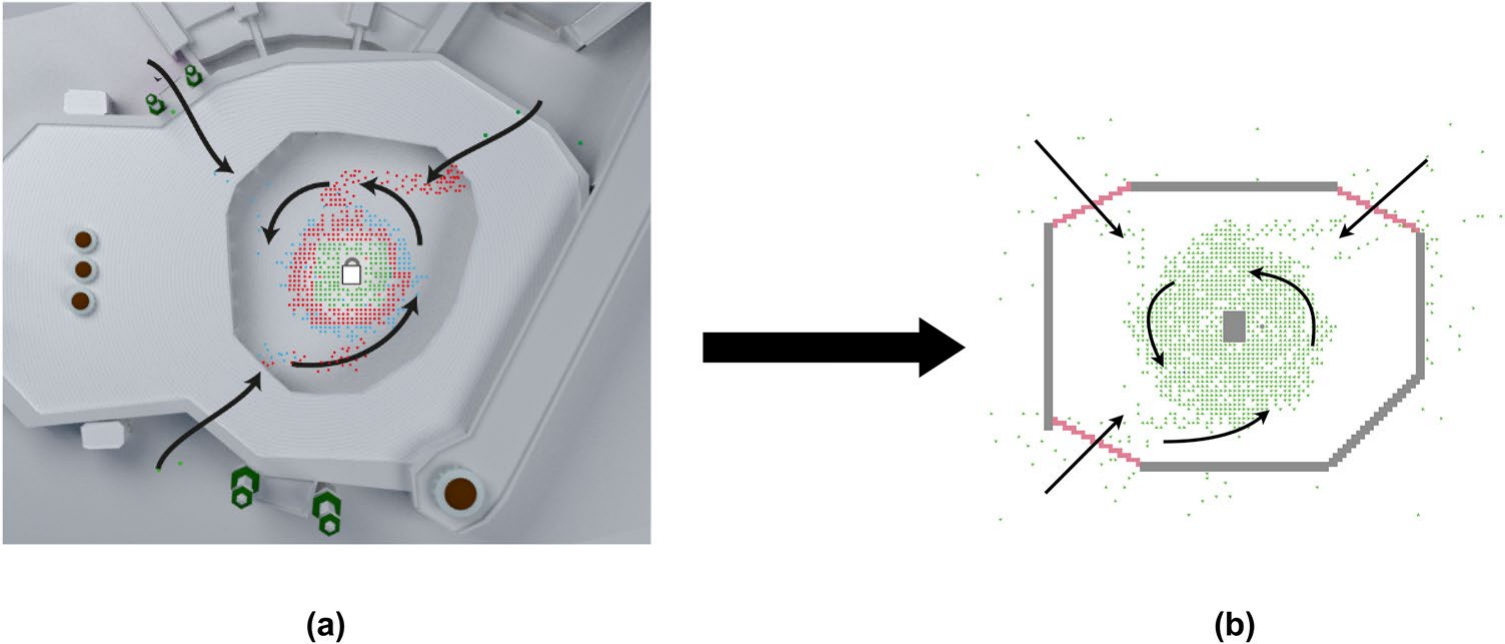
The environment is represented as a grid. The colored dots are simulation indicators representing the agents. (**a**) Overlays the environment model with the geometric blueprint of the Mataf area to highlight the captured spatial structure. (**b**) shows the environment model and crowd dynamics in NetLogo. The overall crowd dynamics are shown using the arrows: agents initially begin outside the Mataf area and aim to move towards the center in a counterclockwise spiral pattern within the spatial constraints of the area.

Randomness is important in order to ensure that we consider a wide range of different scenarios, especially for search-based approaches such as genetic algorithms. We induce randomness in the simulation by assigning random initial positions outside the walls of the Mataf for all agents and by selecting a random movement when several feasible movements are available. Figure 1 shows the developed environment model and the crowd dynamics of the agents in the environment per the behavior rules.

**Table 1:** Description and values of the simulation parameters.

Parameter	Description	Value
$I_0$	Probability of being infected upon contact with an infected agent	0.5%
$N_{0,infected}$	Initial number of infected agents	1
$N_{total}$	Maximum ritual capacity	3000
$(\mu _{v1},\, \sigma _{v1})$	Mean and standard deviation for the distribution of vaccination group 1	(95, 2)
$(\mu _{v2},\, \sigma _{v2})$	Mean and standard deviation for the distribution of vaccination group 2	(70, 2)
$(\mu _{v3},\, \sigma _{v3})$	Mean and standard deviation for the distribution of vaccination group 3	(45, 2)

### Infection and vaccine dynamics

We define the infection dynamics based on two agent classifications: susceptible and infected. The disease is spread upon direct contact between an infected and a susceptible neighbor, i.e. agents on adjacent positions. At each simulation step, an infected agent may infect any one of its susceptible neighbors with a certain probability $I_0$ (see Table 1). Once a susceptible agent becomes infected , they are immediately able to infect others with no latent period. While COVID-19 has been shown to exhibit a non-zero latent period^32^, our model assumption of no latent period represents a worst-case scenario as the spread of disease in the population is overestimated. We also assume that infectiousness is time independent, i.e. constant, due to the short time taken to perform the ritual.

**Figure 2:**
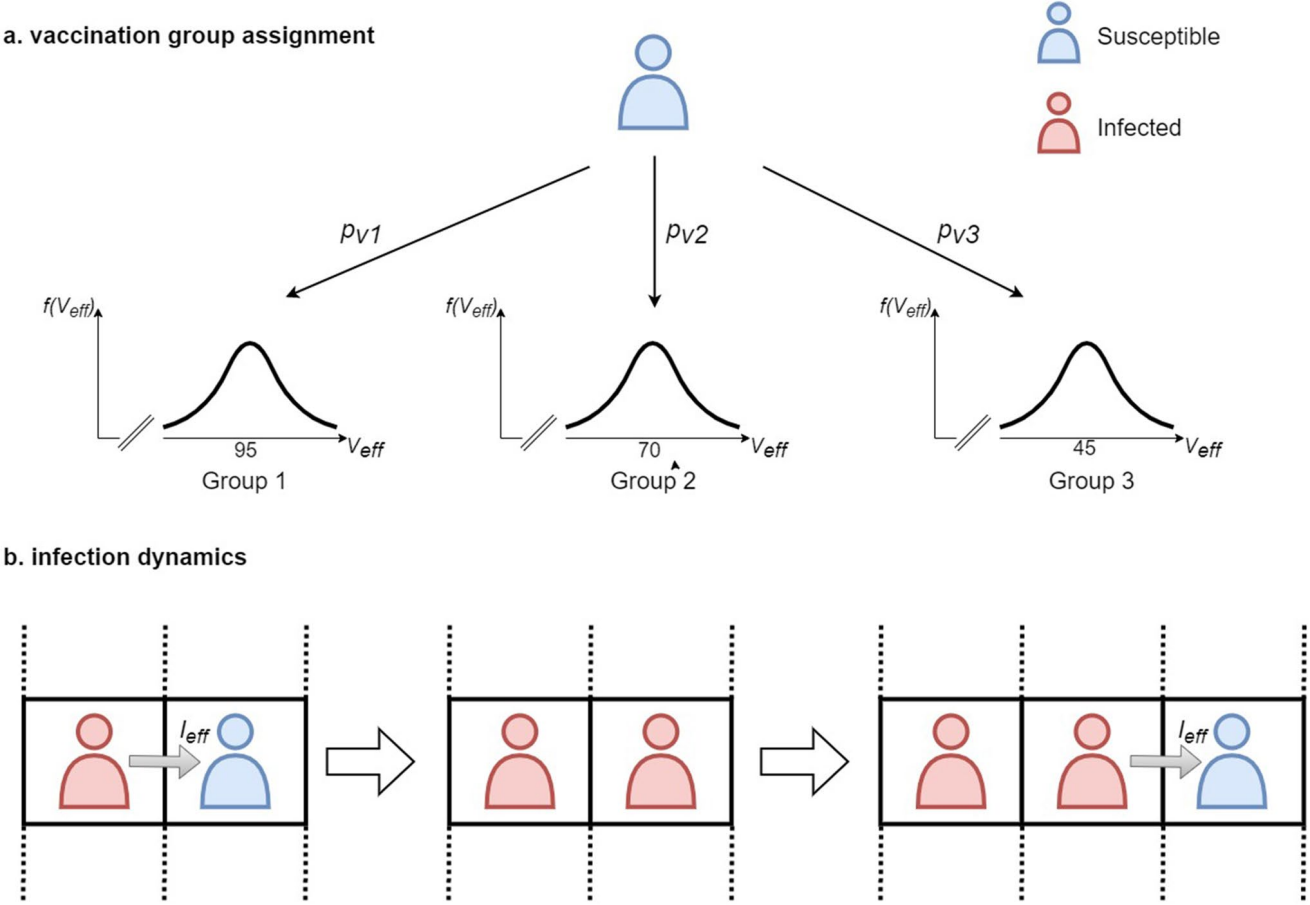
(**a**) shows the assignment of an agent to a vaccination group characterized by the distribution of the vaccine efficacy. Each agent has a predefined probability, $p_{vi}$, of belonging to a vaccine group *i*. The vaccine efficacy, $V_{eff}$, is then sampled from the Gaussian distribution corresponding to that group. (**b**) shows the spread of infection in the population. Infection spreads from direct contact of an infected agent (shown in red) with a susceptible agent (shown in blue) with a probability $I_{eff}$. Once an agent is infected, they immediately become infectious.

Health officials have imposed vaccine requirements on pilgrims attending the Hajj. As such, we will assume that all agents are vaccinated and divide the population based on three vaccination groups. These vaccination groups represent agents with different levels of vaccination. Empirical studies have shown that vaccine efficacy decreases over time and depends on the number of doses received^33–35^. Policymakers can use the following factors to determine the vaccination group of an individual: vaccine type, number of vaccine doses (for multi-dose vaccines), and days since the last dose. Health requirements for the Hajj require that pilgrims share immunization history and such information can be collected and grouped to represent a distribution of the vaccine efficacies, which can be further categorized to the vaccination groups. Figure 2 shows the vaccination group assignment and infection dynamics of an agent in simulation.

Simulated agents are assigned a certain vaccine efficacy, $V_{eff}$, drawn from a Gaussian distribution corresponding to the agent’s vaccination group. Such a distribution can be modeled from empirical data on vaccine efficacy^33,34^. Table 1 gives the parameters for the distribution of each group. The proportion of each vaccination group from the total population is defined at the start of each simulation and determines the probability of an agent belonging to each of the groups. The goal of the simulations is to give insight on the proportion of each group based on certain evaluation metrics to guide policymakers. We then define the probability of being infected upon contact with an infected agent as follows:

1 $$\begin{aligned} I_{eff} = I_0 N_c V_{eff}, \end{aligned}$$

where $N_c$ is the number of contacts with infected agents.

### Genetic algorithm

We model the problem of finding the best control strategy as a continuous genetic algorithm^36^. The algorithm works by randomly initializing a set of solutions (a generation), running the simulation for each solution, and then generating a new set of solutions based on crossover and mutation techniques. This process is repeated until the solution converges or a certain fitness level is reached.

#### States

The state vector defines the set of parameters being optimized by the genetic algorithm. In this case, the proportion of each vaccination group and the total ritual capacity. Let $p_{v1}$, $p_{v2}$, and $p_{v3}$ represent the proportion of the total population from vaccination group 1, 2, and 3 respectively. Moreover, let $N_{pop}$ be the ritual capacity and $N_{total}$ be the maximum ritual capacity. There exists a linear dependence between the proportion of each vaccination group ($p_{v1} + p_{v2} + p_{v3} = 1$) so special consideration is required to avoid unrealistic solutions under this constraint. For example, an increase in $p_{v1}$ needs to be met by a decrease in $p_{v2}$ or $p_{v3}$. To handle this constraint in the genetic algorithm, we instead define the state vector using transformed variables that remove the linear dependence. We define the state vector, under the transformed variables, as follows:

2 $$\begin{aligned} c= \begin{bmatrix} p_1&p_2&p_3 \end{bmatrix}, \end{aligned}$$

where $p_1$ is the proportion of agents in group 2 that are not in group 3, $p_2$ is the proportion of agents from group 3, and $p_3$ is the ritual capacity as a proportion of the maximum capacity. Such a definition removes the dependence constraint between the proportions of each group while still optimizing the parameters of interest: the proportion of each vaccination group and the total ritual capacity. The policy for the distribution of vaccination groups can then be calculated as follows from the state vector:

3 $$\begin{aligned} \begin{array}{ll} p_{v1} &{}= 1 - p_{v2} - p_{v3}\\ p_{v2} &{}= p_1 (1-p_2) N_{pop} \\ p_{v3} &{}= p_2 N_{pop} \end{array}, \end{aligned}$$

where

4 $$\begin{aligned} N_{pop} = p_3 N_{total}. \end{aligned}$$

All state variables are normalized in the range of 0 to 1. Furthermore, we define a minimum ritual capacity of 5% based on the recommendations in the unvaccinated case^16^ and to avoid trivial solutions (0% capacity).

#### Solutions

Each generation consists of a total of $N_p=10$ solutions, where the first generation contains randomly initialized solutions. Subsequent generations need to maintain the best solutions from the previous generation while also generating new solutions to explore the possible states. Each generation contains three types of solutions:

- *Elitist solutions* The best $N_e=2$ solutions from the previous generation are transferred to the next generation.
- *Crossover solutions* $N_t=3$ solutions are selected from the previous generation then the best two solutions, $\{c_1, c_2\}$, are selected to generate a new solution. A randomly selected parameter, $\beta \sim U(0,1)$, is drawn to perform the following crossover function on a randomly selected state variable:
  5 $$\begin{aligned} p_i=\beta p_{i,c_1} + (1-\beta )p_{i,c_2}, \end{aligned}$$
  where $p_i$ is the state variable and $i=1,2,3.$
- *Mutation solutions* The remaining solutions from the previous generations that are not elitists or have had crossover are selected and each state variable, $p_i$, is randomized based on a probability $p_m=0.3$.

#### Fitness function

We model the fitness function based on three factors that would be important for Hajj policymakers when determining an appropriate control strategy:

- *Disease prevalence* This measures the ability of the current control strategy, or solution, to curb the spread of infection in the case of an infection event. We define the disease prevalence, $\eta$, as the ratio of total infected agents to the total population in a simulation run. Note that in this case the ritual capacity represents the total population within the simulation run.
  6 $$\begin{aligned} \eta = \frac{N_{T,infected}}{N_{pop}} \in [0, 1], \end{aligned}$$
  where $N_{T, infected}$ represents the total infected agents at the end of the simulation. $\eta = 1$ represents the entire population is infected.
- *Diversity* This measures the population diversity by looking at the number of people coming from each vaccination group. We measure diversity using the Simpson’s Diversity Index^30^:
  7 $$\begin{aligned} D = \sum _{i=1}^R \frac{n_i(n_i-1)}{N_{pop}(N_{pop}-1)} \in [0, 1], \end{aligned}$$
  where $R=3$ is the number of vaccination groups, *i* is the group index, and $n_i$ is the number of agents in vaccination group *i*. $D=0$ represents infinite diversity in the population and $D=1$ represents no diversity, i.e. all agents come from one vaccination group.
- *Ritual capacity* The ritual capacity, *R*, measures the capacity of ritual used in the current control strategy. We define this as:
  8 $$\begin{aligned} R = p_3 \in [0,1], \end{aligned}$$
  where $p_3$ is the ritual capacity as a proportion of the maximum capacity from the state vector. $R=1$ represents full capacity and $R=0$ represents no capacity. However, in this case, we impose a minimum capacity requirement in the definition of the state vector to avoid the case of not having any agents present.

The total fitness function is then calculated as:

9 $$\begin{aligned} f(\eta ,D,R) = K_1(1-\eta ) + K_2(1 - D) + K_3 R, \end{aligned}$$

and

10 $$\begin{aligned} K_1 + K_2 + K_3 = 1, \end{aligned}$$

where $K_1$, $K_2$, and $K_3$ control the weight of each fitness function component. For example, we can prioritize one aspect of the fitness function such as disease prevalence over the other aspects by tuning these weights, i.e. increasing $K_1$. The goal of the genetic algorithm is to find the solution that maximizes the fitness function in Eq. (9). We frame the problem such that the optimal solution would be one that minimizes disease prevalence, maximizes diversity, and maximizes the ritual capacity.

## Results and discussion

We performed simulations of the genetic algorithm under different weight distributions for 50 generations and the evaluation metrics in the fitness function: disease prevalence, population diversity, and ritual capacity. The weights were distributed to represent scenarios where policymakers might prioritize one, two, or all evaluation metrics. All simulations were initialized with the same seed to ensure similar conditions. We first run a baseline scenario of not implementing any control measures in the Hajj. This represents no conditions on the vaccination status of the pilgrims or the ritual capacity and acts as a comparison for the subsequent experiments. The recorded prevalence for the baseline scenario was 97%, which represents a high rate of infection in the population and further stresses the need for the implementation of effective control strategies.

**Figure 3:**
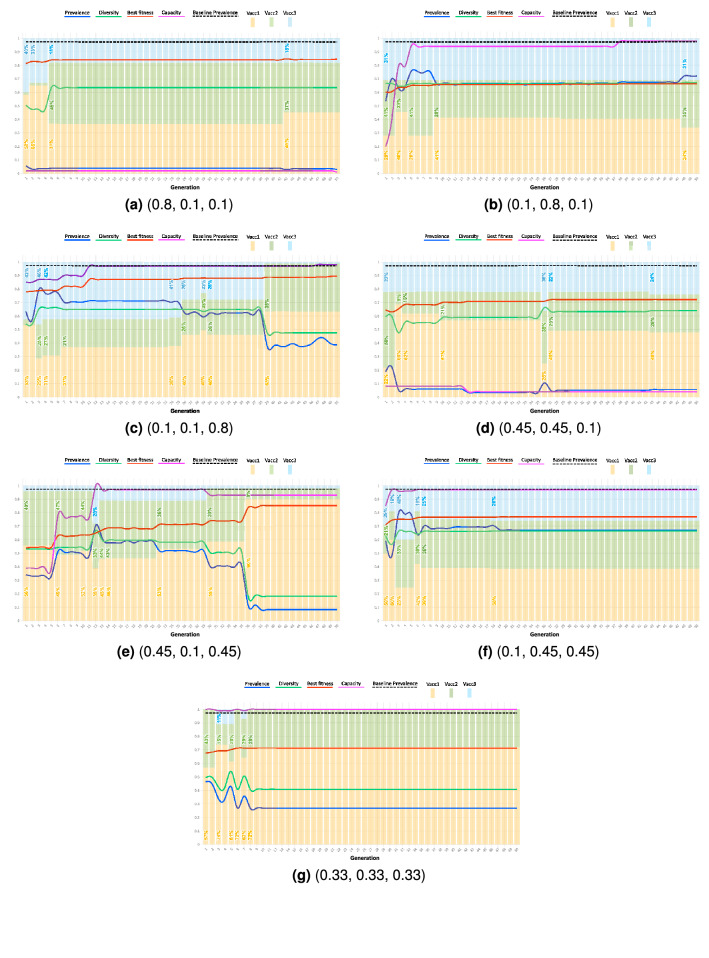
Evaluation of different control strategies under different fitness function weights of the genetic algorithm. The weight of each fitness function parameter from Eq. (9) is given under each plot ($K_1$, $K_2$, $K_3$) which represents the policy objective being optimized. Capacity and vaccine groups are given as a proportion of the entire population. Prevalence is given as a metric calculated from Eq. (6). Diversity is calculated from Eq. (7) and a larger value represents a more diverse population. A trade-off is seen between strategies that focus on decreasing prevalence and increasing diversity. An increase in the population vaccine diversity results in increased prevalence. A focus on decreasing the disease prevalence results in low capacity but still allows for diversity to be present. No clear trends emerge when focusing on capacity alone due to not placing emphasis on diversity and prevalence.

Figure 3 presents the results of these simulations. Prevalence and vaccination groups are presented as proportions of the entire population. Diversity is a metric where a higher value represents a larger population diversity. We summarize the results for the different strategy combinations that prioritize the different evaluation metrics:

- *Prevalence* These strategies focused on drawing pilgrims from high efficacy vaccination groups: Group 1 (mean efficacy = 95%) and Group 2 (mean efficacy = 70%); and limiting the ritual capacity to less than 5%.
- *Diversity* These strategies draw equal proportions of pilgrims from each vaccination group: 34% from Group 1, 35% from Group 2, and 31% from Group 3. However, they resulted in a high prevalence of the disease, approximately 75% of the population was infected. Their contribution towards limiting the spread of the disease is small, however, ritual capacity is near the maximum. High disease prevalence is associated with these strategies.
- *Capacity* These strategies focused on maximizing the ritual capacity with little consideration for diversity and prevalence. Such strategies focused on drawing pilgrims almost exclusively from high efficacy vaccination groups: 63% from Group 1, 36% from Group 2, and 1% from Group 3. High disease prevalence is associated with these strategies.
- *Prevalence and diversity* These strategies focused on having a low ritual capacity, less than 10%, with similar distributions from Group 2 and Group 3 (28% and 24% respectively), but a larger proportion from highly vaccinated individuals in Group 1 (48%). The disease prevalence in this case was reduced to less than 10%.
- *Prevalence and capacity* These strategies focused on selecting individuals primarily from Group 1 (90%) with little diversity from the other groups (8% from Group 2 and 1% from Group 3). The disease prevalence in this case was reduced to less than 10%.
- *Diversity and capacity* These strategies operate the ritual at almost full capacity while almost evenly selecting from each vaccination group. However, with such strategies, the disease prevalence is high (larger than 60%). This still represents more than a 30% decrease in disease prevalence from the baseline case.
- *Prevalence, diversity, and capacity* These strategies give equal emphasis to disease prevalence, population diversity, and capacity. Such strategies select pilgrims from the high efficacy vaccination groups (Group 1 at 72% and Group 2 at 28%) with no pilgrims coming from the low efficacy group. The ritual runs at full capacity and the disease prevalence is lowered to 27%.

Analyzing these results, several trends emerge that describe the efficacy and focus of each control measure. First, strategies that focus on decreasing the disease prevalence prioritize decreasing ritual capacity and selecting pilgrims from high efficacy vaccination groups, the former more than the latter. For example, we notice that for the prevalence case, pilgrims are not exclusively selected from high vaccination efficacy groups, but rather, there is diversity in the population with contributions from groups with lower vaccination efficacy. The decrease in capacity results in a more socially distant gathering and reduces the total number of contacts, which agrees with the trends seen in the disease prevalence and the population diversity. Second, strategies that focus on diversity do not depend on the ritual capacity nor the vaccination groups and lead to generally high prevalence rates. However, the ritual capacity under such strategies can be decreased to decrease the overall prevalence in the population while maintaining a high diversity. Similarly, for such strategies, the diversity can be skewed towards higher vaccination groups to lower the disease prevalence. In this case, prevalence was decreased to less than 10% of the population by biasing selection of pilgrims towards higher efficacy groups. Third, strategies that focus on ritual capacity have varying effects on the diversity and the prevalence rate as the focus is on increasing the number of pilgrims and not selecting pilgrims from specific vaccination groups. For example, if the majority of pilgrims come from higher efficacy groups, then both a lower prevalence and a lower diversity is seen. However, this is not necessarily guaranteed. These trends indicate a clear trade-off between the prioritization of each evaluation metric.

## Conclusion

In this paper, we proposed a tool for the evaluation of different control strategies under three evaluation metrics: disease prevalence, population diversity, and ritual capacity so as to guide policymakers on the effects of different control strategies under the consideration of vaccine inequality. The Hajj ritual was used as a case study as it represents a global mass gathering event. We proposed a genetic algorithm approach to find effective control strategies and employed an agent-based model to capture the spatiotemporal structure of the Tawaf ritual and model the spread of infection. Furthermore, we provided a quantification of the vaccine diversity in the population under each control strategy using the Simpson’s Diversity Index. Simulation results showed a trade-off between the three evaluation metrics when considering effective control strategies. Strategies that focused on decreasing disease prevalence emphasized a lower event capacity while still allowing for vaccine diversity. Strategies that focused on vaccine diversity resulted in high disease prevalence, but that can be addressed by biasing the selection of participants towards groups with higher vaccine efficacy. As such, policymakers should adapt their control strategies as the COVID-19 pandemic progresses to external factors such as the global disease incidence and vaccine adoption; and internal factors such as religious requirements, vaccine diversity quotas, and local health system capacity. During high global incidence, strategies that focus on decreasing the disease prevalence should be emphasized. Whereas with the downtrend of infection and increased adoption of vaccines, strategies that focus on capacity and vaccine diversity can be emphasized. Assumptions were made in the simulation such as overestimating the incidence rate within the population by assuming no latent period between being infected and becoming infectious. This work provided an analysis of control measures that did not implement time constraints on the participants. Future work can consider the effect of introducing scheduling and time constraints based on different vaccination groups to minimize contact.

## Data Availability

The datasets generated during and/or analysed during the current study are available from the corresponding author on reasonable request.
